# Latin Prescriptions

**Published:** 1846-03

**Authors:** 


### LATIN PRESCRIPTIONS.

We think we perceive pretty clear indications that Latin prescriptions, in the United States at least, are going out of vogue. They are still defended by a few, but the great majority of physicians feel that they are a relic of the pedantry of ancient times, when medical books were written, and medical correspondence and examinations carried on in Latin. They are ridiculous; because, as a general rule, they are neither Latin nor English, but a mongrel of the two. They are hurtful to the profession, by maintaining about it that air of mystery and mummery which rendered it, for long ages, the butt of the world. It must be the wish of every sensible man to see it divested of every thing that bears any semblance to humbuggery.

